# Rural-urban transformation shapes oasis agriculture in Morocco’s High Atlas Mountains

**DOI:** 10.1038/s41598-024-81569-7

**Published:** 2025-01-27

**Authors:** Youness Boubou, Kira Fastner, Andreas Buerkert

**Affiliations:** Organic Plant Production and Agroecosystems Research in the Tropics and Subtropics (OPATS), University of Kassel, Steinstrasse 19, 37213 Witzenhausen, Germany

**Keywords:** GIS mapping, NDVI time series analysis, Land use change, Rural-urban disparities, Shifting livelihoods, Sustainable oasis agriculture, Environmental sciences, Environmental social sciences

## Abstract

Traditional agricultural activities and rural livelihoods in Morocco’s High Atlas Mountains are rapidly changing. This is triggered by increasing rural-urban interactions and new livelihood opportunities in cities. A typical example is the oasis of Tizi N’Oucheg in the country’s High Atlas Mountains, which over centuries was largely self-sufficient in food grain and livestock production. Improved infrastructure and better connections to distant urban centers have caused declining livelihood reliance on agricultural activities and enhanced dependence on remittances and the tourism sector in the region. Based on the case of Tizi N’Oucheg, this study aims at assessing the socio-economic and ecological implications of rural-urban transformation for ancient oasis systems in Morocco. Surveys on agricultural practices, census, and meteorological data were combined with GIS (Geographical Information System) -based analyses to examine land use and cropping pattern changes of 625 fields from 1967 to 2022. For the GIS analyses, historical aerial images, multispectral satellite images, and drone-based surveys were used to generate manually classified agricultural fields and NDVI (Normalized Difference Vegetation Index) time series. Our results show a major decline in cultivated land from 13 ha to 6.8 ha over the past 50 years accompanied by an expansion of modern infrastructure since the 2000s. Land management has shifted from labor-intensive multiple cropping and natural fertilization to monocropping of barley for local livestock feeding and increased application of mineral fertilization. The challenging geography of the oasis increased the hardship of practicing traditional agriculture, and therefore largely determines the response of the community to rural-urban transformation. Our data also highlight the increased financial dependence of rural populations on urban centers and the demise of traditional, sustainable agriculture in Morocco’s High Atlas Mountains if policies on agricultural development are not adapted to rural circumstances.

**Supplementary Information:**

The online version contains supplementary material available at 10.1038/s41598-024-81569-7.

## Introduction

From the 1960’s until the 1990’s, Morocco witnessed rapid population growth, together with increasing urbanization processes, which led to major rural-to-urban migration^1–3^. In 2022, 65% of Morocco’s population lived in urban areas compared with 50% in the 1990’s^4^. Given diverse employment opportunities even for unskilled laborers and overall economic development, cities are perceived as gateways to the modern world and financial prosperity^5^. Governmental investments in urban infrastructure, technology, services, and institutions such as health care and education, particularly attracted Morocco’s rural population^6^.

In contrast, rural areas, often characterized by widespread poverty of smallholder farmers who constitute 70% of all holdings, were largely neglected^7,8^. Since the end of the colonial period, structural adjustment programs supported large-scale commercial agriculture in the favorable lowlands to the detriment of mountainous areas with their harsh environmental conditions. Industrial and export-oriented production was subsidized, while subsistence-based agrarian livelihoods were abandoned^9,10^. In many regions of Morocco, rural agricultural production is hampered by complex land tenure regimes, low yields, and limited access to markets^11,12^. Land degradation, desertification, and recurrent droughts pushed most farmers to search for off-farm employment or to rely on remittance payments of migrated family members^13,14^.

Consequently, Morocco’s typical rural livelihoods are rapidly vanishing, reflecting the increasing economic and social disparities between rural and urban spaces. This has particularly severe implications for the traditional, often millennia-old oases agroecosystems in Morocco’s Atlas Mountains. Such settlements are widespread in the country’s southern and eastern regions but currently only host about 5.3% of the national population^15^. Many oases thrived in arid and semi-arid environments through high levels of biodiversity^16,17^, indigenous knowledge on agricultural practices, and a sustainable use of limited natural resources^18,19^. Most oases rely on gravity-fed irrigation systems, refered to as *Souagui* or *Khettara* in Morocco, which channel water harvested through ancient catchments to the fields^20^. The complex multi-layered agroforestry systems of oases that depend on livestock-mediated nutrient cycling are very water efficient and thus also resilient against the effects of climate change^21–23^. Such oases which have stood the sustainability test of time, offer opportunities to better understand the role of social-ecological interactions for sustainable land use systems and the maintenance of rural livelihoods in marginal environments^24^.

Implications of rural-urban transformation and socio-economic changes on agricultural activities have been widely studied at large-scale regional levels using remote sensing and socio-economic data^25,26^. However, assessment approaches adapted to small scale ecosystems and taking into account their special social and ecological features are scarce. This is particularly important as tailor-made rural development measures are required to foster their sustainable future^27^. In addition, remote sensing techniques for monitoring cropping patterns as an indicator of change in agricultural activities using NDVI time series, focused for too long on low resolution multi-year datasets as the Moderate Resolution Imaging Spectroradiometer (MODIS)^28^, Landsat^29^, or Satellite Pour l’Observation de la Terre (SPOT)^30^, while recent high-resolution datasets as Sentinel-2 are rarely used.

Our study aimed at assessing land use and cropping pattern change of a small-scale oasis system in Morocco’s Atlas Mountains using a mixed-method approach. We hypothesized that combining high spatial and temporal resolutions of remote sensing datasets and time series analyses with surveys on livelihoods and agricultural practices can reveal why and how rural-urban transformations change traditional agro-ecosystems. The results may help to make more informed policy decisions for sustainable rural development.

## Materials and methods

### Agroecological setting

#### Geographic context

**Fig. 1:**
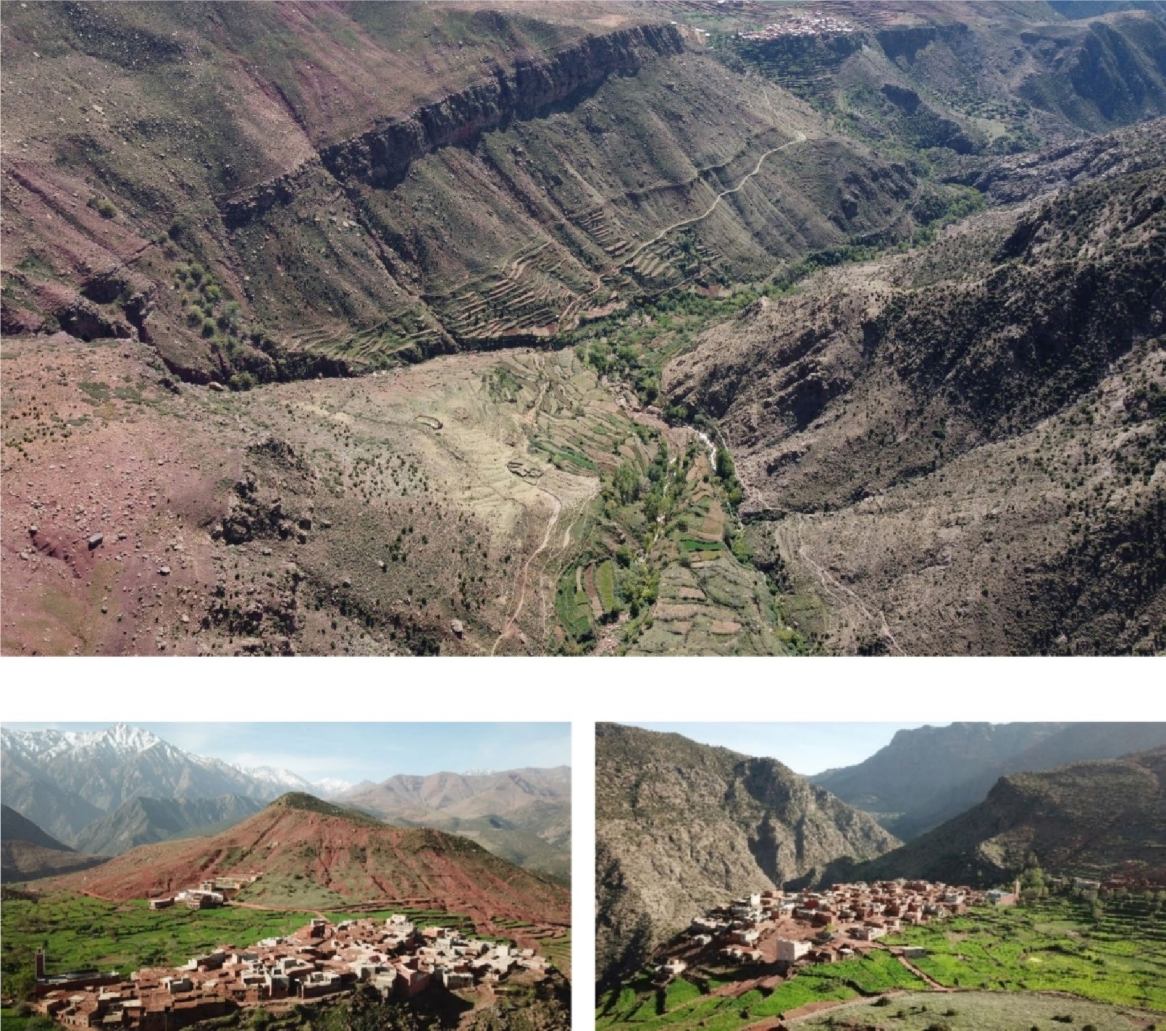
Cropped agricultural terraces and settlement of the mountain oasis of Tizi N’Oucheg, High Atlas Mountains, Morocco.

**Fig. 2:**
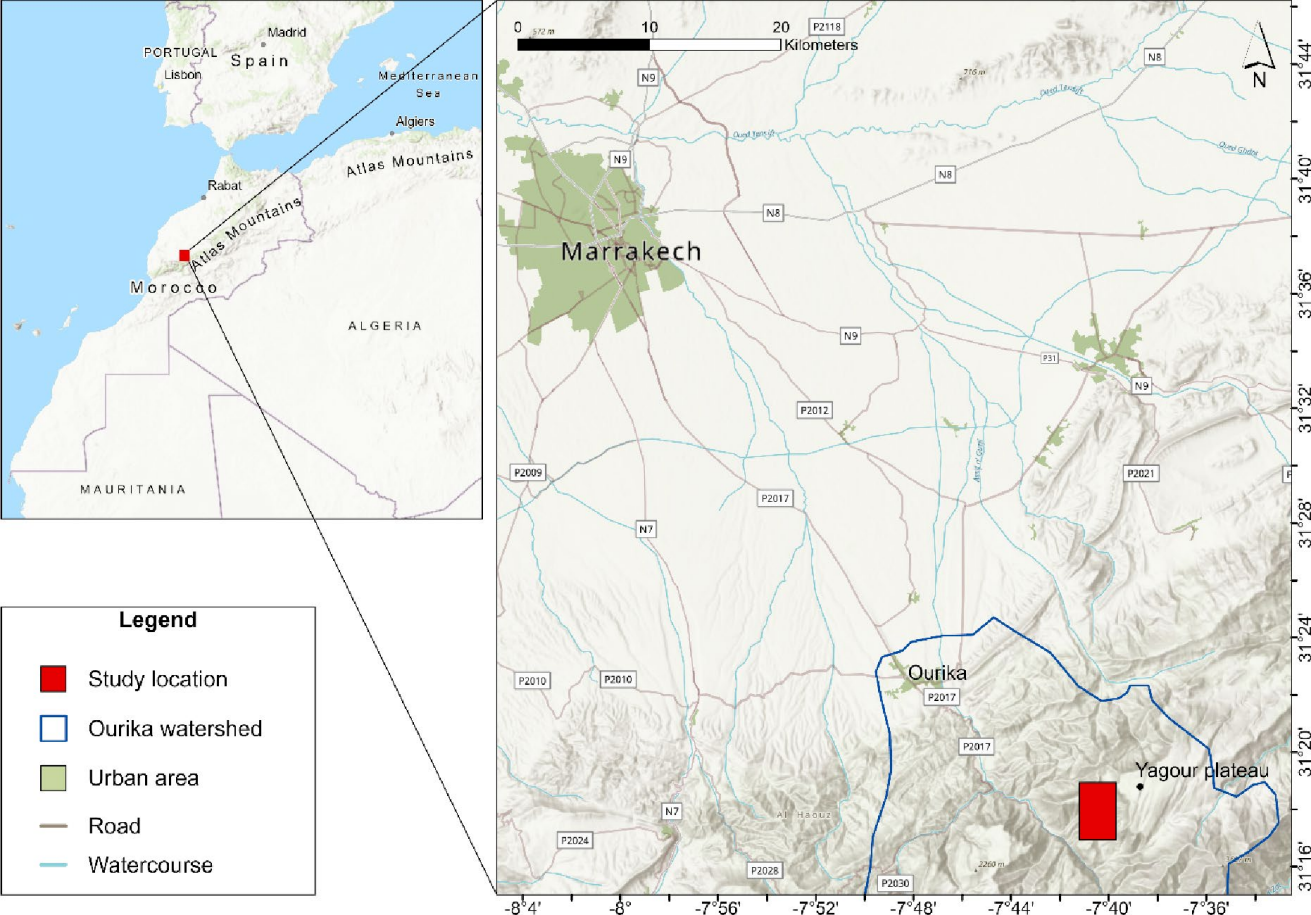
Location of the oasis Tizi N’Oucheg in the Ourika watershed, High Atlas Mountains, Morocco. ArcGIS Pro v.3.1 (https://www.esri.com/arcgis/products/arcgis-pro/); Basemap: Esri, NASA, USGS, GEOMATIC, HERE, Garmin, Foursquare, METI/NASA, FAO, NOAA.

The High Atlas Mountain range, with its highest peak Djebel Toubkal of 4,167 m above sea level (a.s.l.) in central Morocco (31°03′43″N; 7°54′58″W), is the country’s most important geological formation. It constitutes an ecological barrier between the Mediterranean and Saharan climate and harbors a multitude of ancient (agro-) ecosystems^31^. Our study focuses on the 800 years old oasis system of Tizi N’Oucheg (31°17’10.60” N; 7°40’36.40” W) located in the Tachmecht valley, one of the northern affluents of the Ourika watershed. It is situated at 1,650 m a.s.l. and is connected to the closest national road P2017 via a recently constructed unpaved track. Administratively the oasis belongs to the rural commune of Setti Fatma in the region of Marrakech-Safi and has a high touristic potential due to its proximity to the country’s main touristic hub Marrakech (Figs. 1 and 2).

The population of the High Atlas is dominated by *Amazigh* communities who have lived in the region for thousands of years. Although several migration movements and geopolitical transitions such as the Arabian conquest in the seventh and eighth century took place, the *Amazigh* preserve many ancient customs, traditions, and values that are based on practicing oasis agriculture combined with pastoralism of small ruminants.

#### Climatic conditions

A dataset of monthly average temperatures, total rainfall, and rainy days from January 2009 to September 2023 was downloaded from https://www.worldweatheronline.com/ and amended by field records of sun and shade air temperatures using HOBO U23-001 Pro v2 and U14-002 data loggers (Onset Computer Corp., Bourne, MA, USA) set at 30 min record intervals starting from July 2022.

**Fig. 3:**
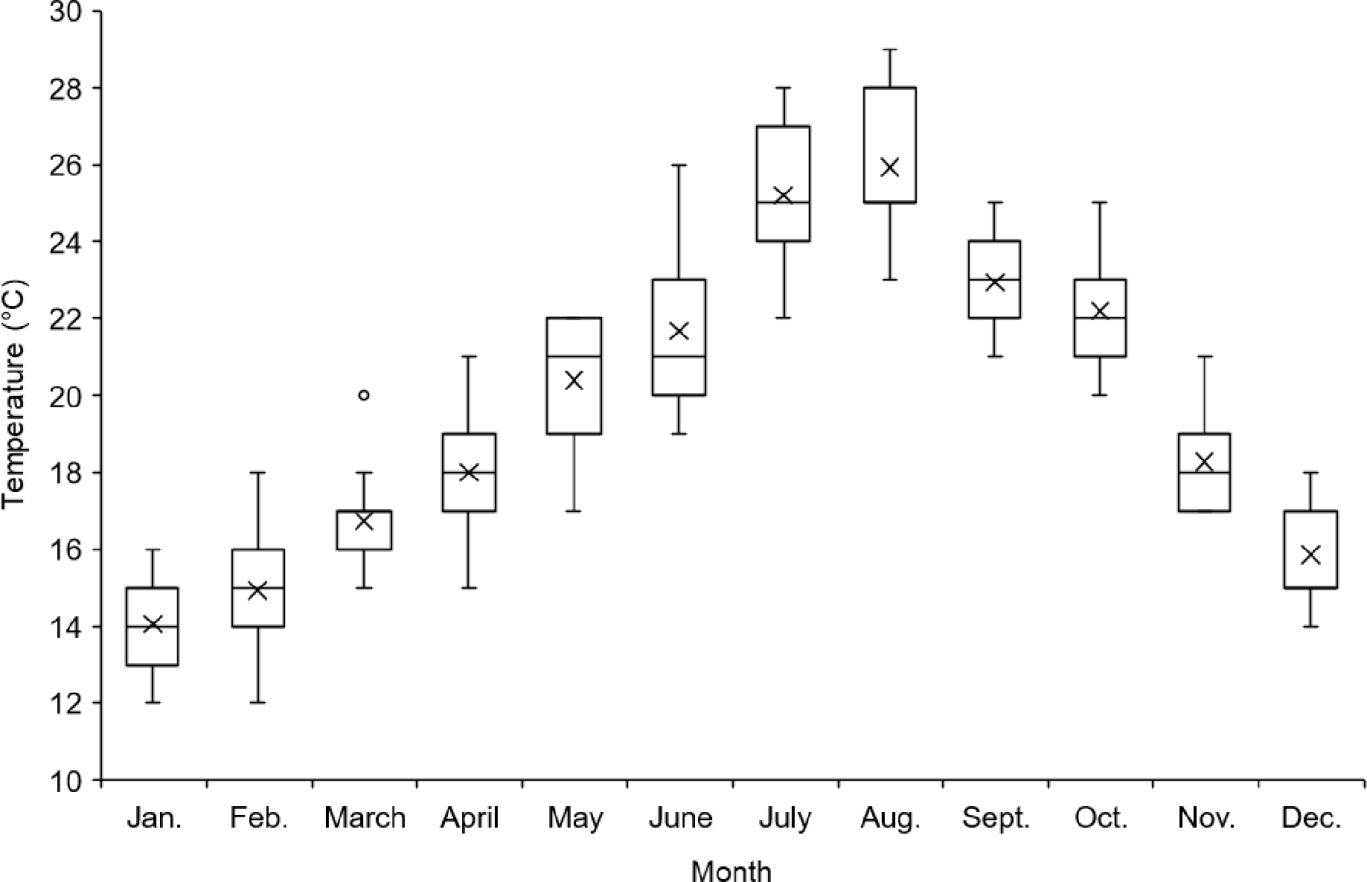
Monthly average temperatures from 2009 to 2023 in the oasis of Tizi N’Oucheg, High Altas Mountains, Morocco (https://www.worldweatheronline.com/).

The climate of the studied oasis is semi-arid Mediterranean with dry summers and cold winters. Average daily temperatures range from 14 °C in January to 26 °C in August with an annual average of 20 °C in the years 2009 to 2023 (Fig. 3).

**Fig. 4:**
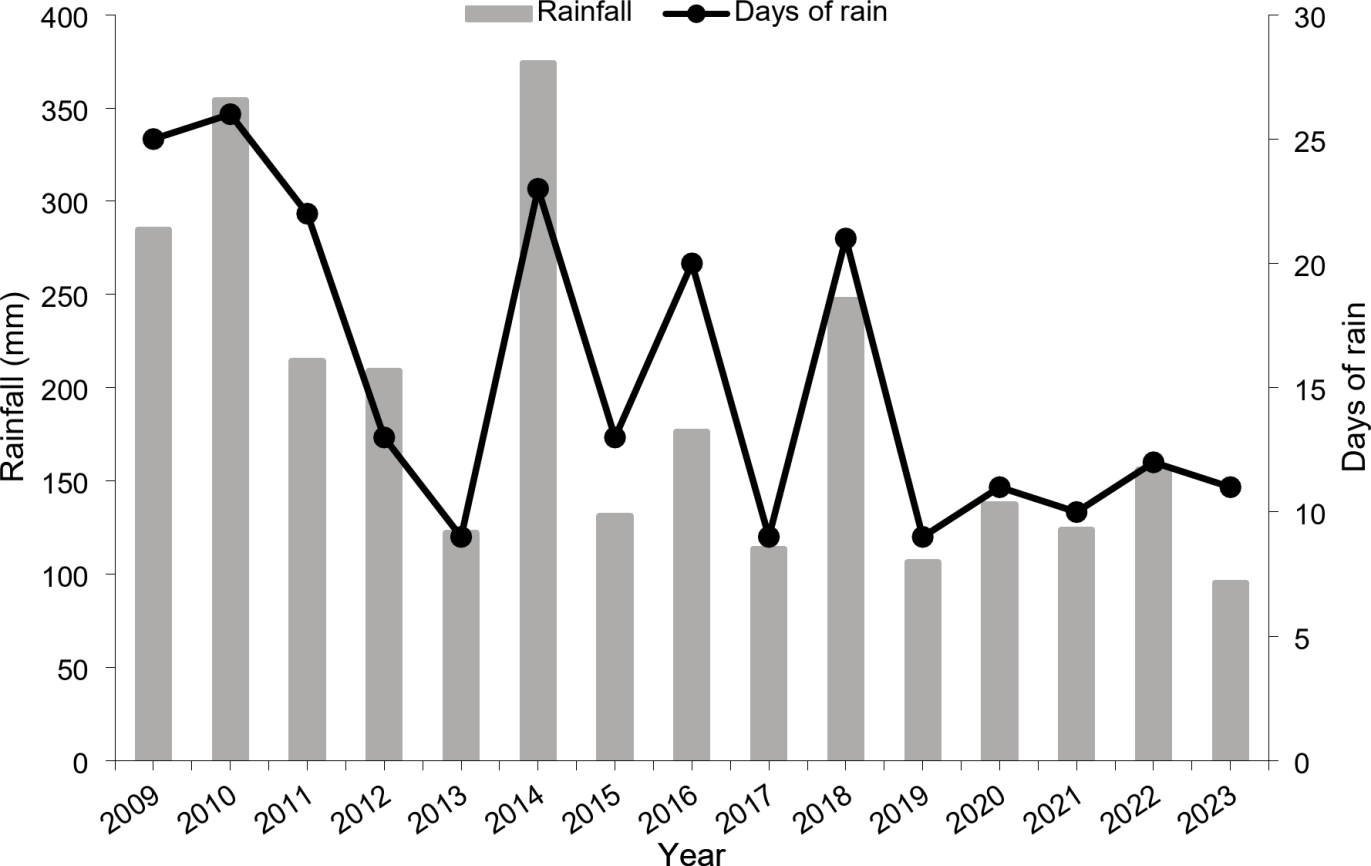
Annual rainfall and days of rain from 2009 to 2023 in the oasis of Tizi N’Oucheg, High Atlas Mountains, Morocco (https://www.worldweatheronline.com/).

The average rainfall recorded in the study area over the last 13 years was 190 mm year^− 1^, distributed over an average of 16 rainy days. The highest annual rainfall of 370 mm was measured in 2014 with 23 days of rain. Dry years with around 100 mm year^− 1^ and not more than 10 rainy days were recorded in 2019 and 2023 (Fig. 4).

The rainy season spans from November to April, with the highest precipitation in February, reaching up to 34 mm. The dry season lasts from May until the end of October, with June, July, and August being the driest months, receiving less than 2 mm of rainfall. Snow usually falls between November and February at an altitude over 1500 m a.s.l^32^.

### Farming system

The first form of land use in the region of Tizi N’Oucheg was likely transhumant pastoralism (2000–3000 BC). For many centuries diverse biomass resources including sparse wetlands and grasslands of wild cereals, xerophytes, and shrubs were preserved in customary protected areas named *Agoudal* which allowed grazing of large livestock numbers. 600 years ago, immigrated families from southern Morocco and from neighboring mountain villages established the oasis settlement of Tizi N’Oucheg and started practicing sedentary crop production on human-made terraces combined with largely pastoral livestock keeping^33^.

Nowadays, irrigated agricultural land stretches over 47 ha whereby cereals, forage, and vegetable crops are cultivated year-round in different cropping schemes. Unlike in desert oases, only few trees are cultivatedas walnuts (*Juglans regia* L.) which grow naturally in terrace hedges and next to water ways. Rainfed terraces were established on narrow mountain slopes but are nowadays often poorly maintained. They largely serve for the extensive production of drought-tolerant cereals such as wheat (*Triticum aestivum* L.) and barley (*Hordeum vulgare* L.).

The irrigated area is organized into districts, with ponds built in their upstreams. Spring water continuously fills these ponds and is afterwards channeled to the fields through traditional gravity-canals. These allow for seepage to foster forage production and tree growth alongside their course. Most of the springs drain rainwater and snow from the *Yagour* plateau which infiltrates in the crevices and clefts of the mountain. Water allocation to agricultural plots is collectively managed and based on customary agreements whereby irrigation rights depend on the area of appropriated land. Goats and sheep are herded in an extensive seasonal transhumance, whereas small numbers of cattle, poultry, and equids are kept inside houses.

### Oasis mapping

#### Land use mapping

**Fig. 5:**
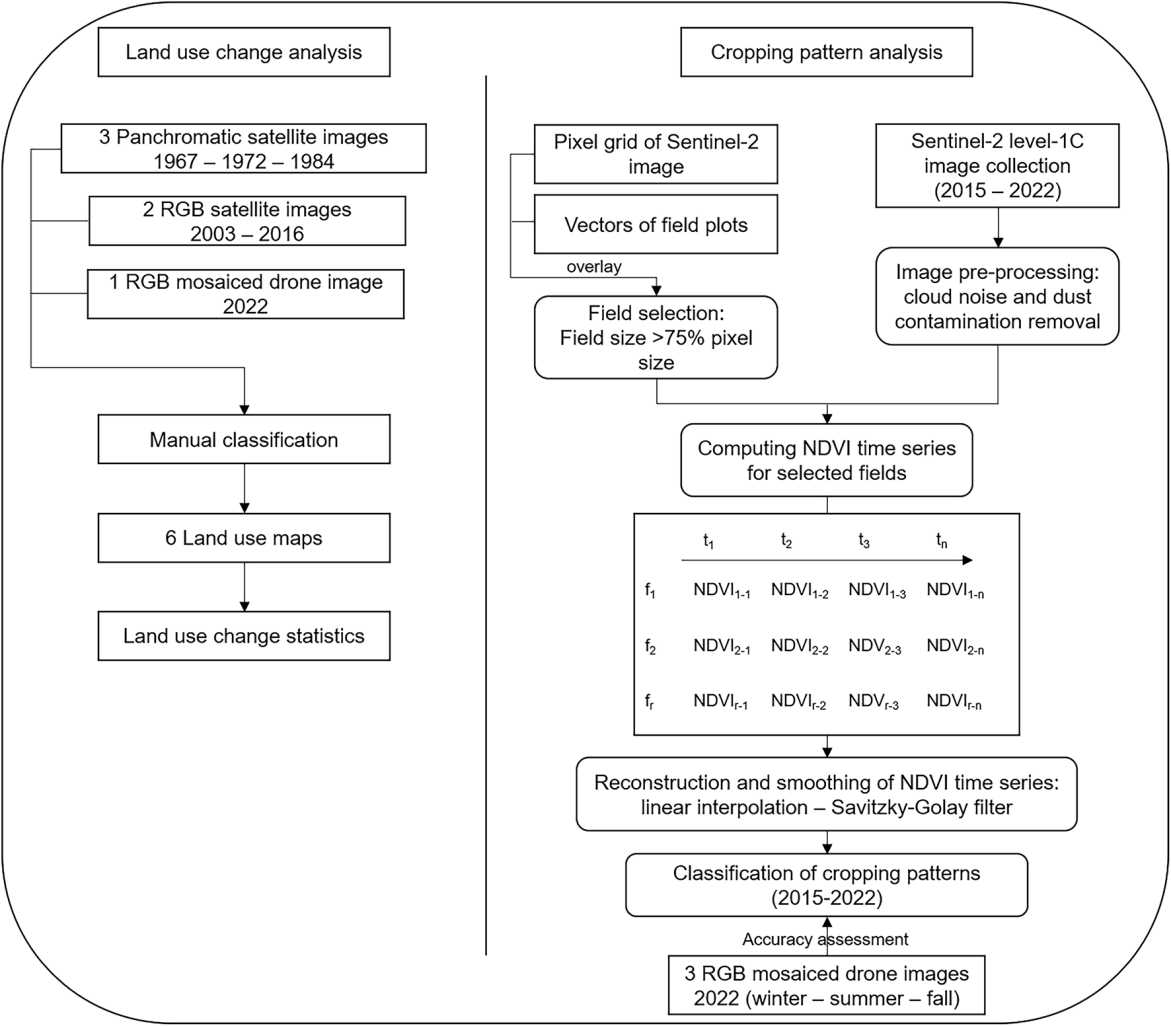
Flowchart for GIS-based analyzes of land use changes and cropping patterns in the oasis of Tizi N’Oucheg, High Atlas Mountains, Morocco.

Land use change (LUC) was analyzed by classifying a high-resolution set of black and white Corona KH-4 images from the 1960s, 1970s, and 1980s^34^, a Quickbird basemap from 2003, an Airbus One Atlas basemap from 2016^35^, and an orthomosaic of drone images from 2022 (Table 1; Fig. 5).

**Table 1:** Remote sensing data for land use and crop analysis in the oasis of Tizi N’Oucheg, High Atlas Mountains, Morocco.

Sensor/Data type	Date	Spatial resolution	Data source
CORONA KH-4 A/Panchromatic	13/05/1967	2.7 m	USGS, EarthExplorer [34]
CORONA KH-4B/Panchromatic	26/05/1972	1.8 m	
CORONA KH-9/Panchromatic	01/08/1984	1.2 m	
Quickbird / RGB	01/05/2003	1.5 m	Google Earth, CNES [35]
Airbus One Atlas/RGB	01/07/2016	< 1 m	Google Earth, Maxar Technologies [35]
Sentinel-2 A, -2B/Multispectral	5 days interval; 01/11/2015–31/10/2022	10 m	Google Earth, ESA [35]
Drone DJI Mavic air 2/RGB	01/04/2022	< 1 m	Personal data

The classification comprises three land use classes: cultivated fields, uncultivated fields, and built-up area. Ground truth data for 125 field plots were collected during field work in 2022 using a handheld Global Positioning System (GPS) Garmin eTrex 30x. Participatory mapping of agricultural land use was conducted with farmers older than 50 years by showing them printed maps, and asking for the verification of classified historical images. This improved the accuracy of the classification and provided a detailed description of change processes in agricultural land and built-up areas.

Given the large diversity of images, we classified all datasets manually to have a comparable scale of measurement and identical geometries between images with different properties^36–38^. For the CORONA grayscale images, reflection and absorption characteristics were used to differentiate between the highly absorptive vegetation and moisture of irrigated land represented by dark pixels, and the highly reflective dry bare land represented by white pixels^39^.

All remotely sensed data and participatory maps were processed using the open-source Quantum Geographic Information System software (QGIS) version 3.22^40^.

### Crop mapping

#### Computing NDVI time series for selected agricultural fields

Changes in cropping patterns were analyzed by mapping the occurrence of crops growing in the oasis. This was based on the built-in Harmonized Sentinel-2 Multi Spectral Instrument Level-1 C satellite images from 2015 to 2022 on the Google Earth Engine platform (GEE). This dataset offers high spatial (10 m) and temporal (5 days) resolution, harmonized radiometric and geometric corrections as well as an orthorectification (Table 1). Atmospheric noise of clouds and dust contaminating the images were removed using two indicators^41^: The general cloud content in every image, which was set to 30%, and the built-in *QA60* bitmask band of 60 m resolution, which masks pixels based on a binary dataset of opaque and cirrus cloud content. NDVI^42^ time series were computed for all selected fields (Fig. 5).

**Fig. 6:**
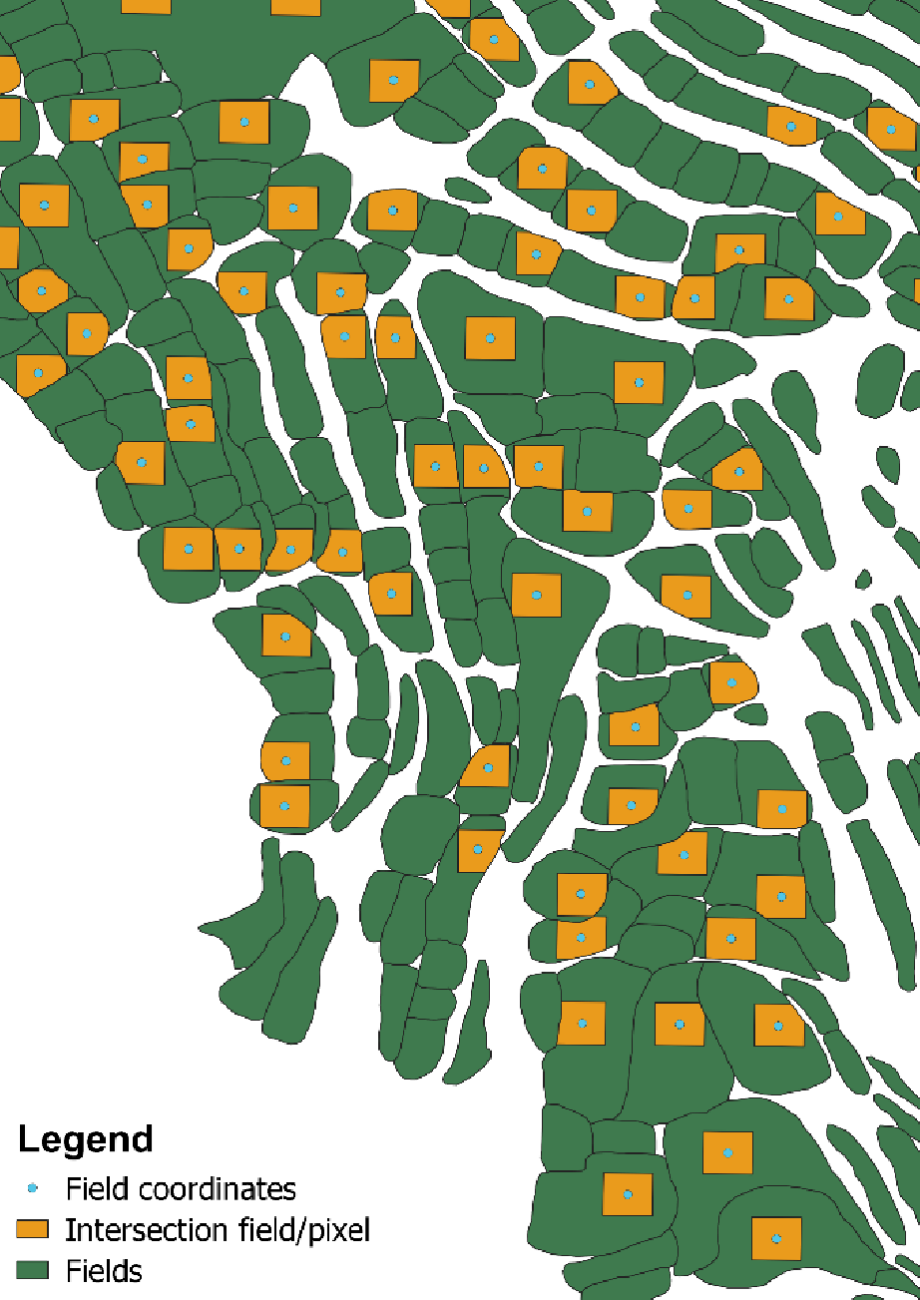
Example of the intersection between Sentinel-2 pixels and vectorized fields in the oasis of Tizi N’Oucheg, High Atlas Mountains, Morocco. QGIS v.3.22 (https://qgis.org/).

Agricultural fields in mountain oases stretching over rough and hilly terrain generally have irregular structures and geometries which constraints two-dimensional mapping^43^. To align with the 10 m resolution of Sentinel-2 images in the relatively small oasis fields averaging 188 m², a filter was applied to retain the fields overlaying with at least 75% of the area of one pixel. This threshold ensures that spectral characteristics of the field content are abundant enough in the case of interference in mixed pixels^44^. In cases where a single field overlayed with multiple pixels, all pixels were retained and their values were averaged in further processing steps. To do so, a sample Sentinel-2 image covering the area of interest was downloaded from GEE and transformed to a grid of pixels in QGIS. The intersections between the grid and the manually digitized fields were generated, their areas calculated, and only the ones over 75 m² were kept. Centre points were generated for every selected pixel and transferred to GEE as field coordinates (Figs. 5 and 6). The total amount of 2,511 fields was reduced to 624 which could be used for further analyses. To evaluate if this field selection does not influence their spatial representativity of the oasis, a correlation analysis between the geographical position of a field and its size was conducted, and showed no significant results.

#### Reconstructing and smoothing NDVI time series

To compute and interpret phenology metrics from NDVI profiles, smoothing time series is necessary to overcome the biased values creating local variations^45^. The Savitzky-Golay approach (S-G)^46^ is a commonly applied method as it usually performs well in the case of negatively biased noise^41^, and if the time series follows a constant growth and decline pattern^45^.

The S-G approach is applicable to sequential data points that have a fixed and uniform interval along a chosen axis, and the data points must form continuous and relatively smooth curves^46^. For that reason, a linear interpolation was applied to create a 5-days equally distanced time series, whereby the data voids from the original time series were replaced by linearly interpolated values (Fig. 5). Values of the closest unmasked pixels within a time window set to 45 days were used to compute the interpolated value^45^. The linear interpolation equation can be expressed as:

$$\:{N}_{i}={N}_{x}+({t}_{i}-{t}_{x})\frac{({N}_{y}-{N}_{x})}{({t}_{y}-{t}_{x})}\:$$

where $\:{N}_{i}$ is the $\:i$th interpolated NDVI value with its respective date $\:{t}_{i}$. $\:{N}_{x}$ and $\:{N}_{y}$ are the closest non-flagged NDVI values and $\:{t}_{x}$ and $\:{t}_{y}\:$are their respective dates.

Savitzky and Golay introduced a simplified method for smoothing and calculating derivatives of consecutive values, which involves a convolution process that is conceptualized as a weighted moving average filter^46^. The weights are determined by a polynomial of a specific degree and perform a polynomial least-squares fit within a defined window when applied to a signal. The purpose of this polynomial is to maintain higher order characteristics of the data while minimizing the bias introduced by the filtering process. The simplified least-squares convolution equation for smoothing NDVI time series can be expressed as:

$$\:{{Y}_{j}}^{*}=\frac{\sum\:_{i=-m}^{i=m}{C}_{i}{Y}_{j+i}}{N}$$

where $\:Y$ represents the original NDVI value, $\:{Y}^{*}$ is the resulting NDVI value after smoothing, $\:{C}_{i}$ corresponds to fixed coefficients depending on the $\:i$th NDVI value in the smoothing window, $\:N$ equals the size of the smoothing window represented by $\:2m+1$, and the index $\:j$ refers to the position in the original data table.

The S-G function was directly applied to the image collection as it is available in the Open-Source Earth Engine Library (OEEL) in GEE (Fig. 5). Two parameters were determined based on the characteristics of the NDVI observations: half the width of the smoothing window $\:m$, and the degree of the smoothing polynomial $\:d.$ Larger $\:m$ values yield a smoother output but may diminish sharp peaks, and larger $\:d$ values reduce filter bias but may lead to overfitting and a noisier outcome. Therefore, the moving window width was similarly defined as the time window of 45 days used to look for unmasked NDVI values, in order to make use of the real NDVI values rather than the interpolated ones, whereby the degree of the smoothing polynomial was set to 3.

#### Classification of the cropping pattern

**Fig. 7:**
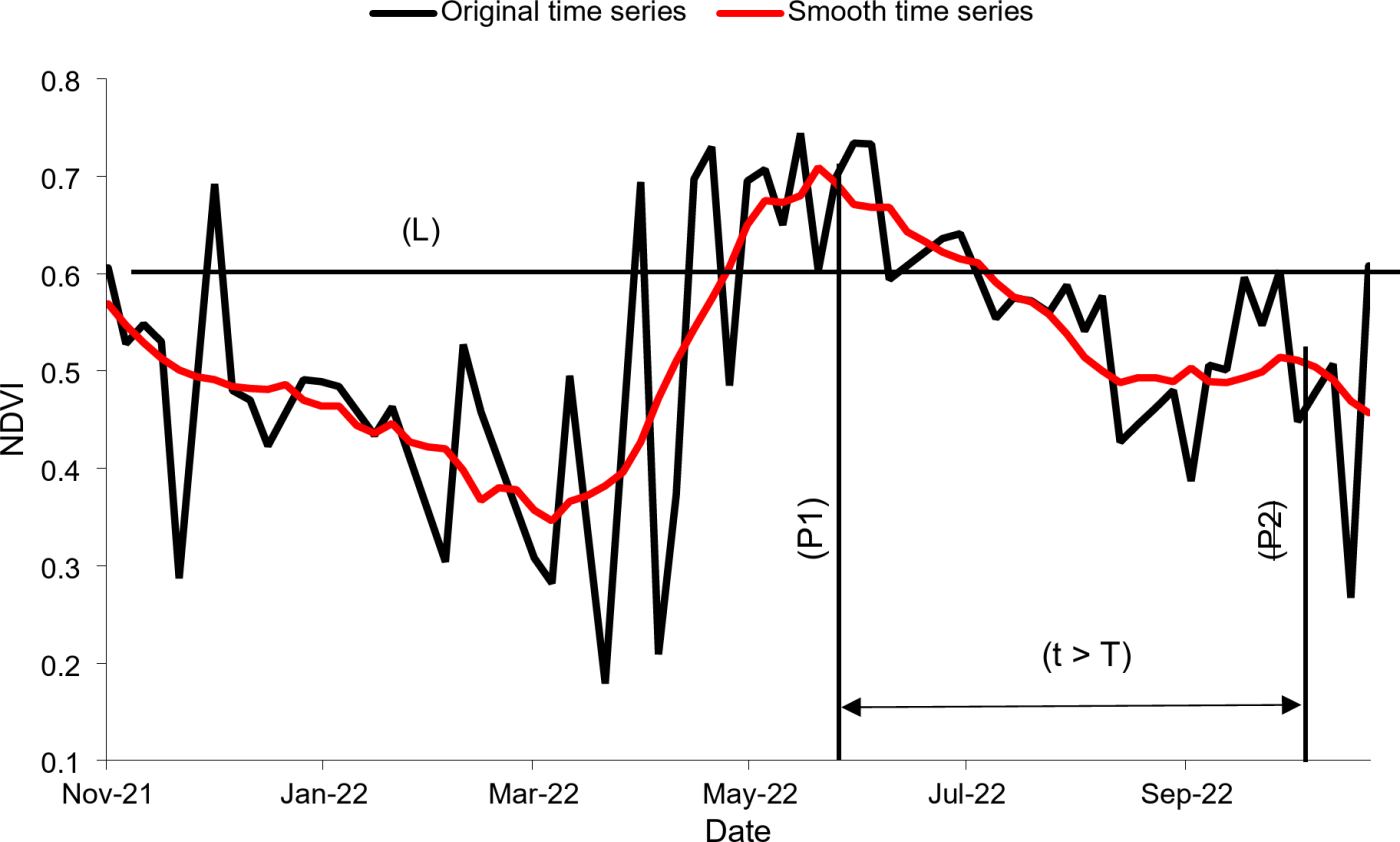
Representation of the NDVI (L) and time (T) thresholds for selecting true NDVI peaks on the smoothened time series in order to map crops in the oasis of Tizi N’Oucheg, High Atlas Mountains, Morocco.

Two cultivation patterns were defined for the classification: monocropping and multiple cropping patterns (sequential cropping; Fig. 5). Fields where no crops were detected, were classified as uncultivated fields. Multiple cropping in the oasis of Tizi N’Oucheg refers to sequential cropping rather than intercropping. Some of Tizi N’Oucheg’s farmers tend to divide their fields into smaller field units cultivated with different crops. In this case, the number of crops in a cropping sequence may be over-estimated due to overlapping NDVI peaks. Therefore, a time threshold (T) of 45 days was defined using the crop calendar as the minimum period between consecutive crops in the oasis, and successive overlapping peaks were downgraded to only one (Fig. 7). To remove false peaks associated with slight changes in soil or plant moisture^47^, an NDVI threshold (L) was set by visually assessing NDVI time series of 20 fields. The threshold was defined as the mode of the real NDVI peak values rounded down to their nearest tenths, which was equal to 0.6 (Fig. 7). To keep consistent time periods for the identification of crop growth cycles, the starting point of an agricultural season was defined as 1 November of every year, and the end as 31 October. The number of crops grown in every agricultural season were retrieved from the smoothed NDVI time series by identifying peak NDVI values using the Findpeaks function from the Practical Numerical Math Functions (Pracma) package in the open source RStudio version 4.2.2 statistical software.

#### Accuracy assessment

To assess the accuracy of the cropping pattern classification, the numbers of crops in each field generated from the classification of the 2021/2022 agricultural season were compared with the number of crops generated from three orthomosaics of drone images recorded in the same period (Fig. 5). A confusion matrix was computed, and the overall accuracy calculated. Given the high resolution of the drone images, cultivated crops were detected, and subsequently the cropping sequence defined for every field. The accuracy was concluded similarly for other agricultural seasons, since the methodology was uniformly applied on the dataset. 

#### Analyses of spatial variations in cropping patterns

To better understand how the spatial location of fields positions in the oasis affect their cropping patterns, a 3D model was generated by combining the GMTED2010 Digital Elevation Model (DEM)^34^ and the classification of cropping patterns. This allowed to determine three spatial characteristics of fields: the altitude of a field, the walking distance from the oasis settlement to a particular field, and the bee-line distance from a field to the respective irrigation pond. Statistical analyses comprised a one-way variance analysis (ANOVA) to test for differences in the spatial characteristic between fields with different cropping patterns by comparing their mean values. A multinomial logistic regression indicated the strength and the direction of the relationship between the spatial characteristics and fields with a certain cropping pattern.

### Survey data

To investigate changes in crop and livestock production as well as in the socio-economic status of farming households, a semi-structured survey was conducted with 45 farmers of Tizi N’Oucheg (Supplementary material 1). The farming households were asked about their household composition, migration activity, income sources, property rights, land use, cropping patterns, field inputs and outputs, as well as livestock numbers. Questions on perceived changes over the past 10 and 20 years in each of these survey themes were also included. The answers were analyzed descriptively and combined with results of the remote sensing analysis.

### Ethical authorization

The study was authorized by the laboratory of Geosciences affiliated to the Faculty of Science Ain Chock Casablanca in collaboration with the University of Kassel, Germany. The data collection procedure and subsequent use of the data was approved by the Central Ethics Committee of the University of Kassel, Germany. Subsequently, informed consent was obtained from all participants.

## Results

### Land use change

**Fig. 8:**
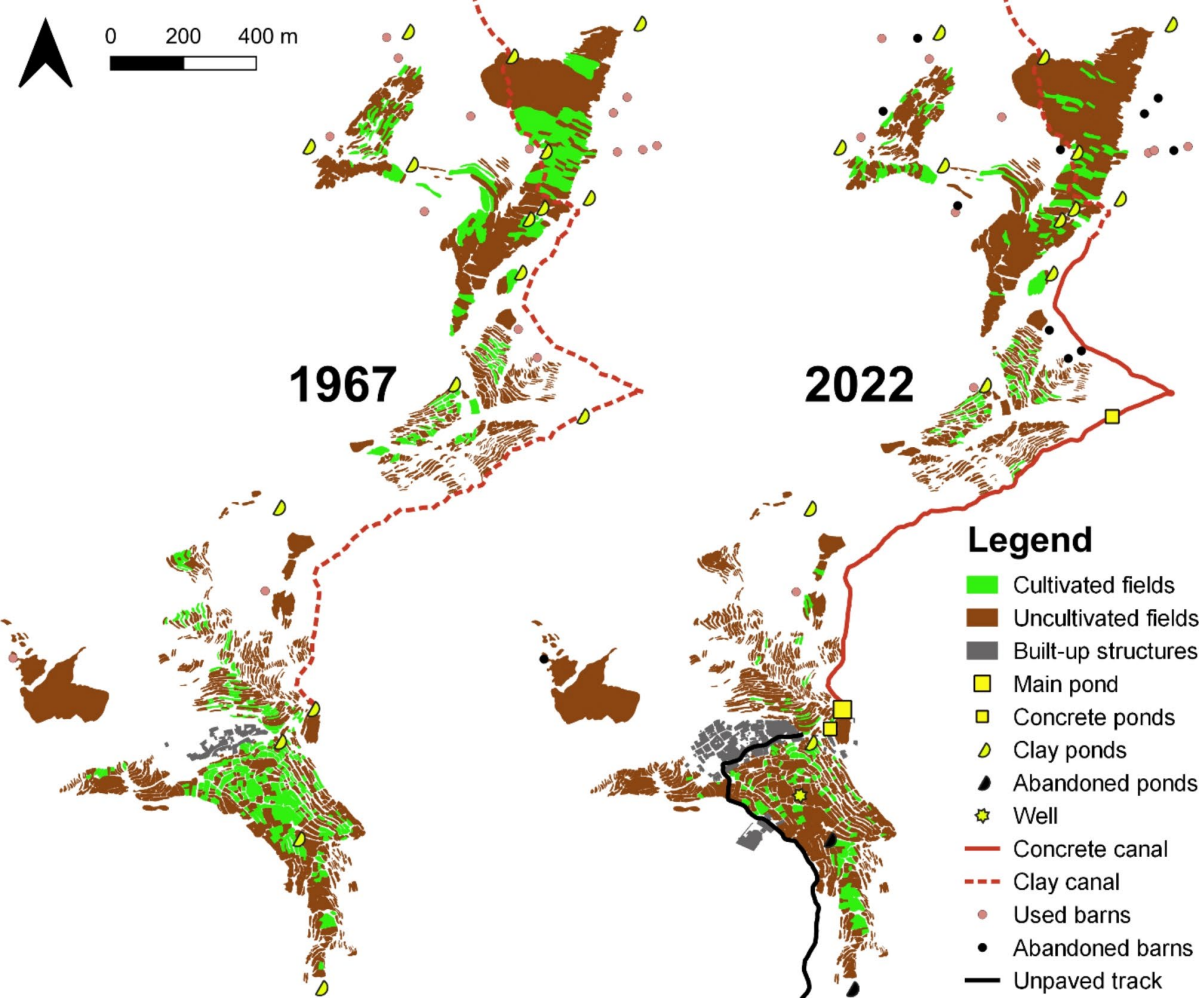
Land use changes from 1967 to 2022 in the oasis of Tizi N’Oucheg, High Atlas Mountains, Morocco based on manual image classification. QGIS v.3.22 (https://qgis.org/).

Analyses of LUCs from 1967 to 2022 showed a decline in the total cultivated area from 13 ha to 6.8 ha whereby this decrease was with 15%, the largest between 1972 and 1984 (Table 2; Fig. 8). Most surveyed households (68%) reported a one third decrease in the cultivation of their total fields over the past 20 years. Main reasons mentioned were lack of irrigation water (52%), insufficient labor force in the household (47%), and the fragmentation of fields to small and scattered units after heritage. Many livestock barns along the Tachmecht valley have become abandoned over time and at present only 10 out of 21 remained operational (Fig. 8). Most households (68%) reported that a decrease in feed production and consequently milk for newborns was a major factor for the decreasing livestock keeping activities (Table 3).

**Table 2:** Changes in land area of cultivated fields and built-up infrastructure from 1972 to 2022 in the oasis of Tizi N’Oucheg, High Atlas Mountains, Morocco.

Year	1967	1972	1984	2003	2016	2022	Δ (2022 − 1967)
Cultivated fields (ha)	13.02	11.87	10.01	8.51	7.12	6.78	− 6.24
Built-up structures (ha)	0.76	1.11	1.52	2.07	2.41	2.84	+ 2.08

**Table 3:** Changes in the % of households keeping animal species between 2002 and 2022 in the oasis of Tizi N’Oucheg, High Atlas Mountains, Morocco.

Animal species	% of households	
	2002	2022
Sheep	88	36
Goat	84	76
Cattle	76	65
Equid	48	20
Poultry	4	8

**Fig. 9:**
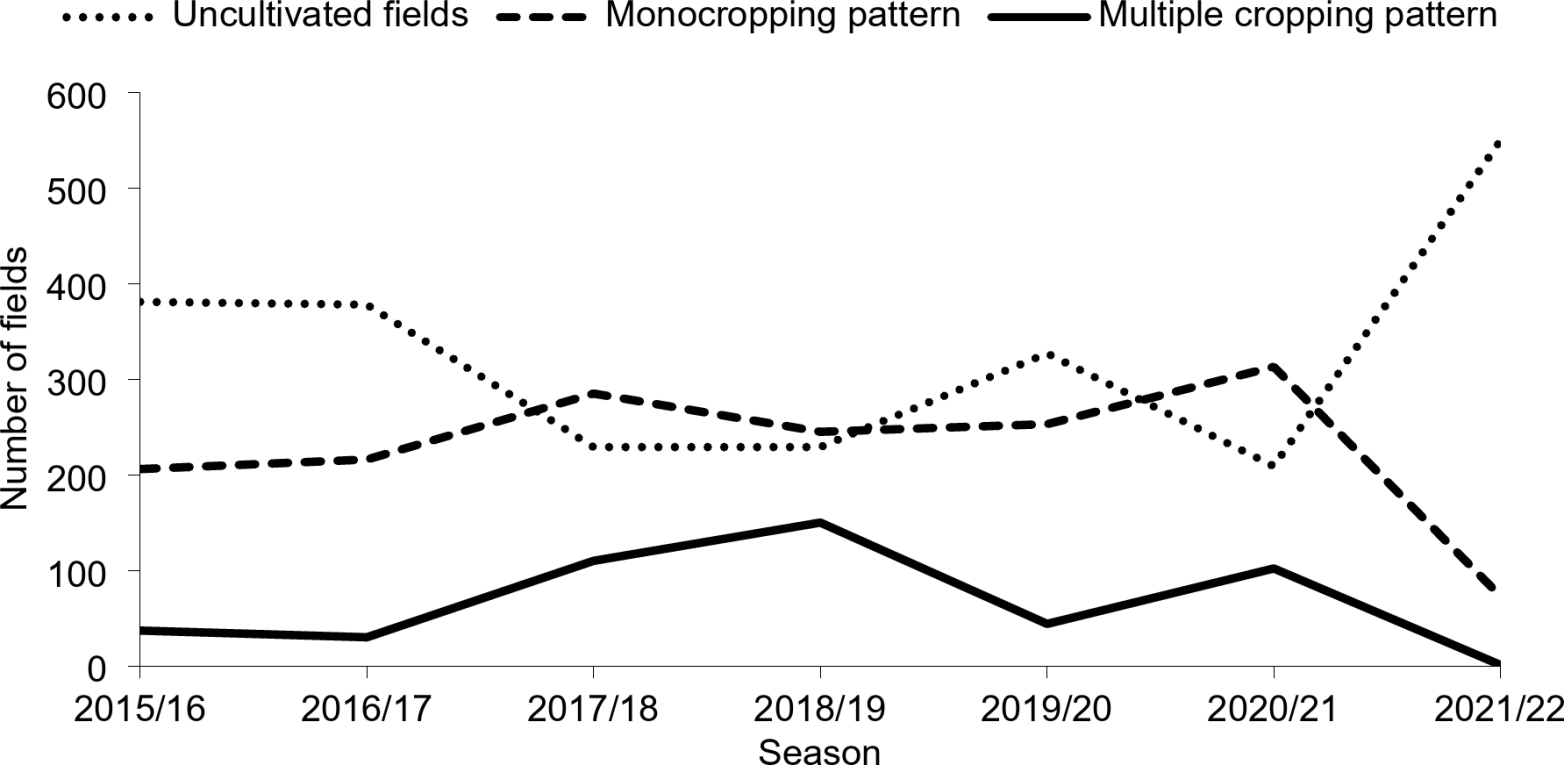
Cropping patterns from 2016 to 2022 in the fields of the oasis of Tizi N’Oucheg, High Altas Mountains, Morocco.

During the same time period the built-up area quadrupled. Since the 2000’s, and particularly after the construction of an unpaved road which eased transport, Tizi N’Oucheg underwent major transformation processes. The location of the oasis and its connection to the Ourika Valley have ensured its prospects as a touristic destination. Investments through non-profit organizations and remittances from former villagers allowed the construction of modern concrete houses, educational and leisure infrastructures, and the establishment of a village-based touristic accommodation. Electricity and potable water infrastructure through a central distribution system combined with the maintenance of old clay-stone houses improved living conditions of the inhabitants (Table 2; Fig. 8).

Over the past 40 years, the irrigation system was subject to restoration with a focus on the supply of water to fields nearby the settlement. Three ponds irrigating fields close to the settlement were reconstructed in 2006 and 2018 from stone-clay to concrete hereby raising the effectiveness of water storage. Two thirds of the primary canal channeling water from the far mountain springs to the main pond of the settlement were rebuilt with concrete in 2006. In 2016 for the first time a well was drilled in one of the fields close to the settlement to a depth of 100 m, allowing access to additional groundwater resources. At the same time, three ponds situated further away from the settlement in the downstream of the valley were abandoned (Fig. 8).

### Changes in farming practices

#### Cropping patterns

Cropping sequences in the oasis of Tizi N’Oucheg are based on four major groups of crops: autumn cereals and legumes such as barley, clover (*Trifolium alexandrinum* L.), and alfalfa (*Medicago sativa* L.), winter/spring vegetables such as potato (*Solanum tuberosum* L.), onion (*Allium cepa* L.), and garlic (*Allium sativum* L.), summer vegetables such as tomato (*Solanum lycopersicum* L.), bell pepper (*Capsicum annuum* L.), eggplant (*Solanum melongena* L.), and summer maize (*Zea mays* L.; Tables 4 and 5). Farmers usually start their cropping sequence at the beginning of the year by cultivating a single crop which may be amended by a second and a third one. Crop cultivation may also start only in spring or summer. In this case it is limited to monocropping or double cropping. Farmers choice for different cropping patterns largely depends on water for irrigation and labor.

**Table 4:**
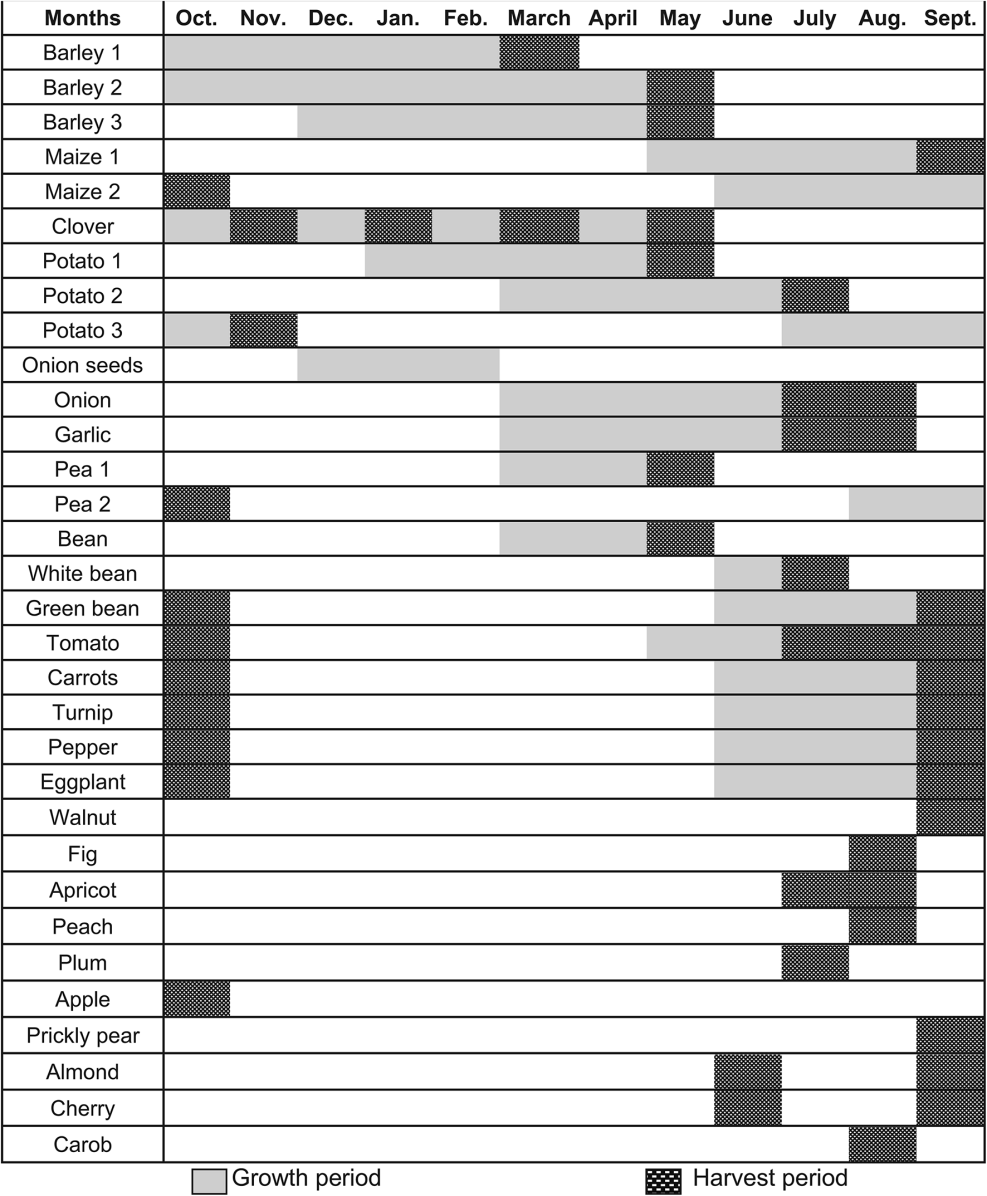
Growth cycles of crops cultivated in the oasis of Tizi N’Oucheg, High Altas Mountains, Morocco.

**Table 5:**
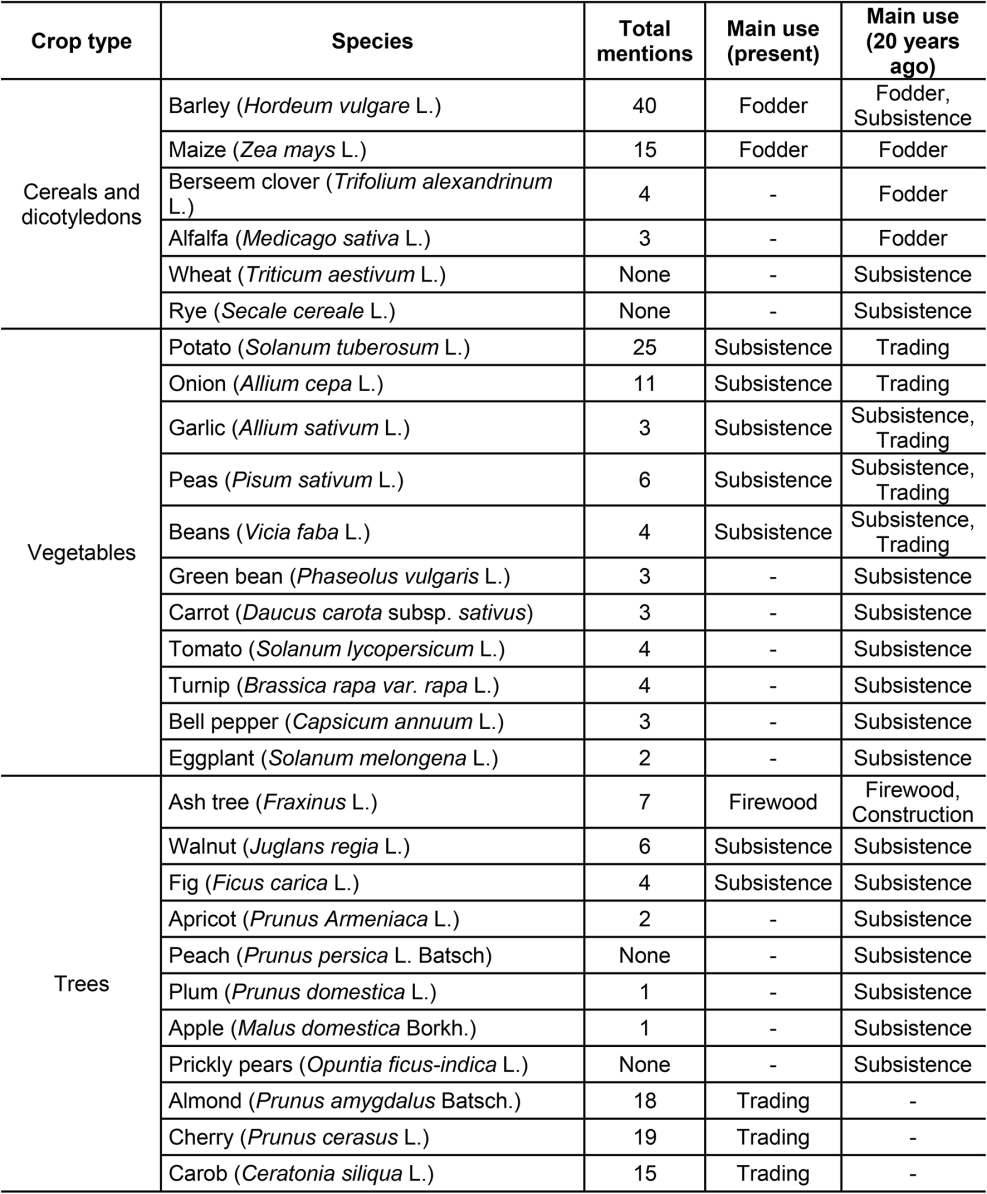
Overview of crop species mentioned by surveyed households, and their past and present uses in the oasis of Tizi N’Oucheg, High Altas Mountains, Morocco.

Cropping patterns, which were determined by mapping crop occurrence at an accuracy level of 61%, were inconsistent in monocropping and multiple cropping from 2016 to 2022. Over the past seven years, monocropping was the major cultivation pattern whereas multiple cropping occupied only 11%, and more than half of the observed fields remained uncultivated (Fig. 9). Most surveyed households (63%) saw the main reason in a water deficit which hindered the cultivation of multiple crops during the summer period. Monocropping predominated during the rainy season (average of 55% compared with 44% in spring and 1% in summer) to benefit from direct rainfall rather than irrigation only.

**Fig. 10:**
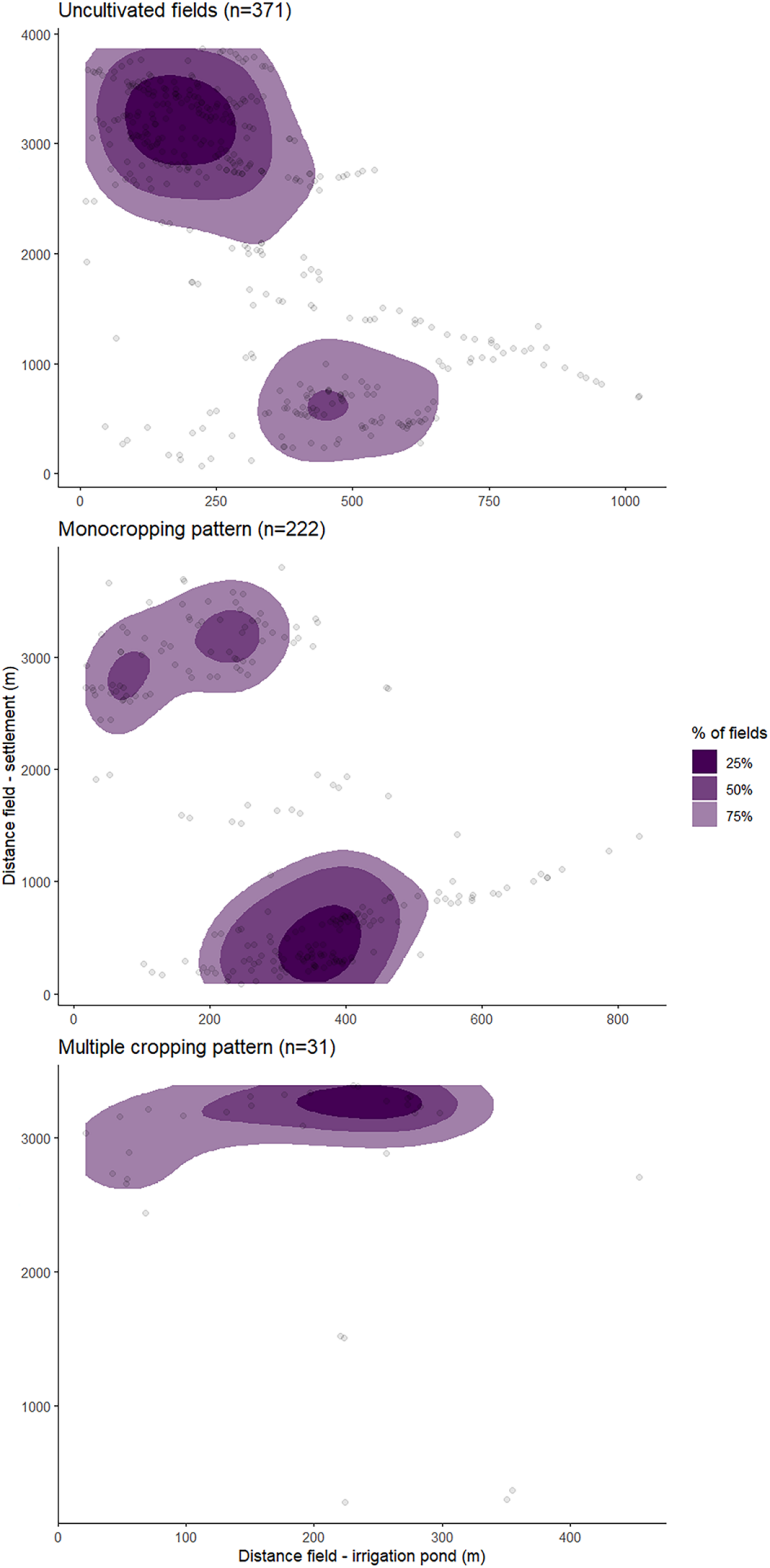
Density plot of fields in gradients of the walking distance from the field to the settlement and the bee-line distance from the field to the irrigation pond. The contour lines define the quartiles of the total field number in every cropping pattern.

**Fig. 11:**
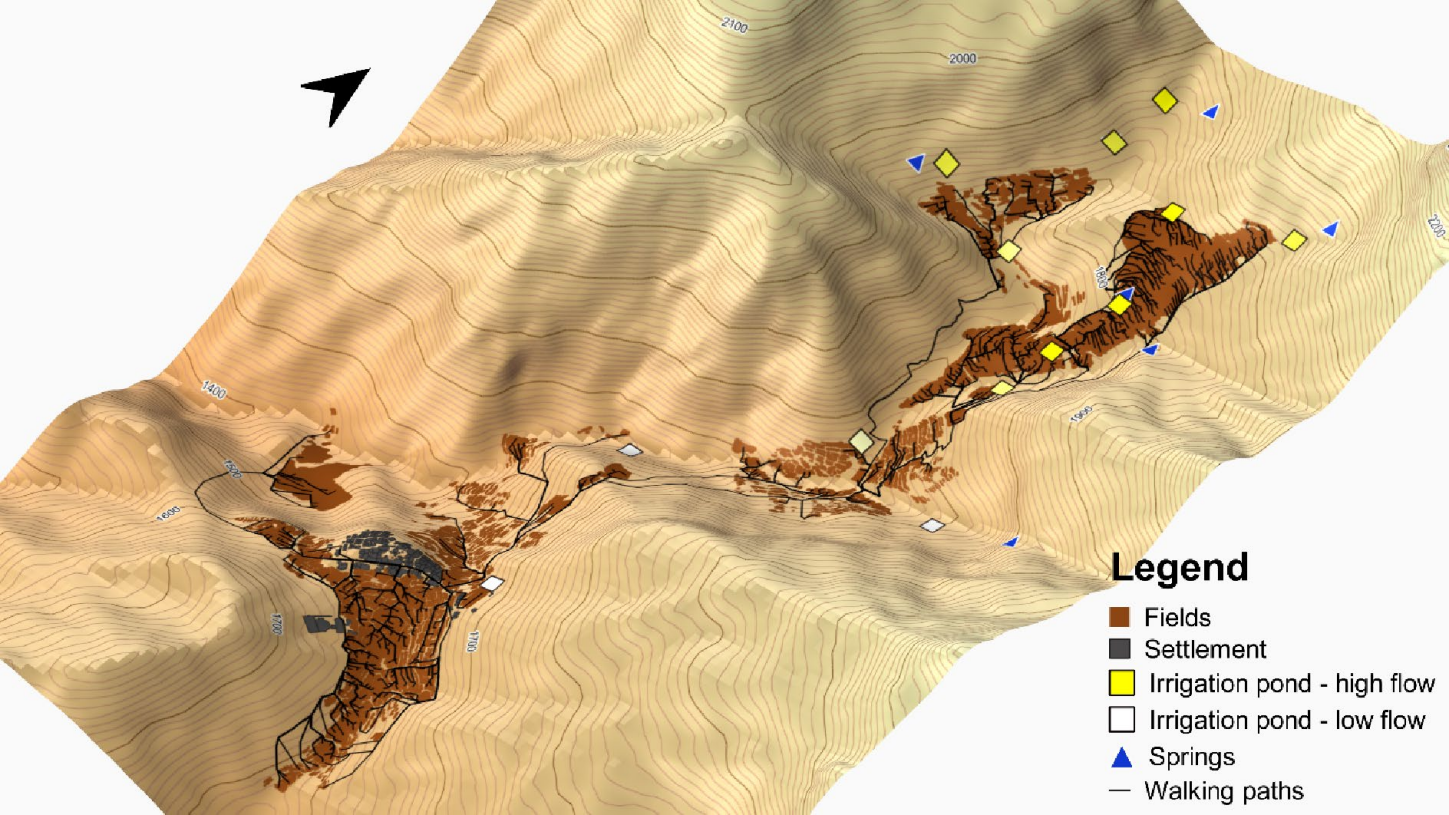
3D model displaying the walking paths, and water flow levels of the irrigation ponds in the oasis of Tizi N’Oucheg, High Atlas Mountains, Morocco. QGIS v.3.22 (https://qgis.org/).

Cropping patterns of fields changed depending on the distance of fields to the settlement or to irrigation ponds (*P* < 0.005 respectively). Over the past seven years, multiple cropping was often practiced in fields far from the settlement and close to irrigation ponds, where water flow was high enough to reach the fields even during the dry season. On the other hand, monocropping was mainly practiced on fields close to the settlement, and did not seem to depend on the existence of irrigation ponds. The effects of altitude on the distribution of cropping patterns in the oasis were similar to the field-to-settlement distance since field altitude and field-to-settlement distance were highly correlated (*P* < 0.005). This highlights the challenging access to the fields in the oasis which always requires long walking hours on the steep slopes of the valley to reach remote terraces (Figs. 10 and 11).

### Diversity and use of crops

Crop cultivation in the oasis is nowadays largely limited to barley as an autumn cereal, and potato and onion as winter/spring crops. Barley satisfied subsistence needs of households 20 years ago but became the major fodder crop to sustain livestock during non-grazing winter periods. In the past, potato and onion were sold to markets but are nowadays mainly cultivated for subsistence while only minor quantities are traded. Former fodder and subsistence crops such as winter legumes and summer vegetables are vanishing, and the cultivation of wheat and rye (*Secale cereale* L.) was abandoned. Similarly, walnut, apple (*Malus domestica* Borkh.), and stone fruits such as apricot (*Prunus Armeniaca* L.), peach (*Prunus persica* L. Batsch), and plum (*Prunus domestica* L.) are no more harvested. Instead, new cultivars of almond (*Prunus amygdalus* Batsch), cherry (*Prunus cerasus* L.), and carob (*Ceratonia siliqua* L.) trees were introduced by recent development projects and are cultivated inside the fields in a rudimentary agroforestry land use system (Tables 4 and 5).

### Crop fertilization

Crop fertilization comprised mineral and organic fertilizers. Typically, manure of goat, sheep, and cattle is applied and thoroughly mixed with the soil before the preparatory tillage, while urea ammonium sulfate (33N-0P-0K) and urea (46N-0P-0K) fertilizers are often used at the sowing stage as a basal application or as a top dressing for established crops. Over the past ten years, the quantity of organic amendments for the main cultivated crops decreased by 30% due to labor scarcity for transporting manure from the far-away livestock barns to the fields. Therefore, in multiple cropping systems the frequency of fertilization was reduced from one application per crop to one application per agricultural season, which benefits less the spring and summer vegetables as it is applied at the onset of the year. Consequently, stacks of manure were left unused and subject to mineralization and subsequent losses through volatilization and leaching.

More than half of the surveyed households used mineral fertilizers for all crop types instead of manure, as it can be applied conveniently before rainfall. Traded crops are subject to intensive fertilization to satisfy customer demands for product size and color. During the last ten years, the quantity of mineral amendments for onion and potato has decreased by 40% because of relatively high purchase prices and low investments in farming activities. Subsequently, crop yields reportedly declined by 20% for barley, 44% for onion, and 33% for potato (Table 6).

**Table 6:** Changes in fertilizer amendments and yields of main cultivated crops over the past 10 years.

Type of fertilizer	Urea (kg ha^− 1^)		Urea ammonium sulfate (33-0-0) (kg ha^− 1^)		Manure (t ha^− 1^)		Yield (t ha^− 1^)	
Time	10 years ago	Now	10 years ago	Now	10 years ago	Now	10 years ago	Now
Barley	708.87 ± 5.41 (650–829)	405.6 ± 6.21 (338–484)	540.11 ± 2.37 (500–573)	532.89 ± 7.68 (415–641)	4.84 ± 0.17 (3–7)	4.04 ± 0.16 (2–6)	5.09 ± 0.33 (1–9)	2.51 ± 0.25 (1–8)
Onion	557.07 ± 6.38 (432–627)	324.29 ± 6.51 (218–427)	487.51 ± 6.47 (387–576)	289.69 ± 5.77 (213–384)	5.04 ± 0.17 (3–8)	2.6 ± 0.14 (1–4)	17.89 ± 0.49 (10–24)	10.33 ± 0.44 (4–17)
Potato	809.84 ± 4.78 (733–876)	524.11 ± 7.75 (437–664)	617.96 ± 5 (552–696)	523.04 ± 2.73 (485–564)	6.84 ± 0.35 (0–12)	3.29 ± 0.29 (0–9)	29.78 ± 0.14 (28–32)	20.62 ± 0.79 (8–31)

Data show annual means and their standard errors and ranges as reported by 45 surveyed farmers in the oasis of Tizi N’Oucheg, High Atlas Mountains, Morocco.

### Changes in livelihoods

Over the past 50 years, livelihoods in the oasis have transitioned from subsistence-based agriculture and local trading of products to increasingly involve off-farm activities. This shift began following the end of the French and Spanish-Portuguese colonial regimes, which occupied Moroccan territories in 1956 and 1975, respectively. The re-creation of the Moroccan state had a significant impact on oasis systems, as out-migration increased in response to the high demand for construction labor in urban centers (Table 7). There was a significant negative correlation (*r* = 0.98; *P* < 0.005) between the area of cultivated fields and the number of migrated households, underscoring the influence of recent migratory movements on farming activities, particularly land cultivation.

**Table 7:** Approximative population and migration numbers from 1970 to 2020 in the oasis of Tizi N’Oucheg, High Atlas Mountains, Morocco.

Year	1970	1980	1990	2000	2010	2020
Population number	240	263	372	423	503	522
Out-migrated households	6	13	32	45	53	57

Apart from fully migrated households, 87% of surveyed households now had at least one member, typically the male household head or an adult son, engaged in seasonal or full-time professions outside of the oasis. Seasonal employment usually lasted 6 to 9 months and was commonly pursued in urban centers such as Casablanca, Kenitra, Rabat, or Marrakech, the latter accessible by daily commuting. These jobs, predominantly in construction, retail, tourism, and catering services sectors, do not require prior qualifications, and contributed with over 50% to the total household income.

## Discussion

The results of our LUC analysis in Tizi N’Oucheg from 1967 to 2022 show a continuous decrease in land cultivation, accompanied by an expansion in the built-up area since the 2000s, reflecting major social-economic changes. Fields classified as uncultivated comprise abandoned and fallow fields. Hereby fallow land refers to cultivated land that remains unused for agriculture for less than five years. If the fallow period is longer, fields are considered as abandoned even if that may not always be true^48,49^. The distinction between long-term fallow and abandoned land is difficult to determine using remote sensing. Nonetheless, the increasing number and time span of fields left uncultivated over the past 50 years clearly shows the diminishing importance of agricultural activities.

The interviewees stated a lack of labor force due to out-migration as a key reason for decreasing land cultivation. Our data show a strong negative correlation between the area of cultivated land in the oasis and out-migration numbers over the last 50 years. Out-migration has also caused changes in livelihood strategies in the oasis as remittances from migrated family members nowadays account for a large proportion of the households’ incomes. Therefore, agriculture as a main livelihood strategy has lost in importance^50,51^.

Additionally, the Atlas Mountains have witnessed increasing touristic activities since the 1980’s with diverse implications for its inhabitants^52^. International visitors coming to Marrakech have started exploring the rural hinterlands for hiking activities^53^ and for their rich cultural heritage^54^. For inhabitants of Tizi N’Oucheg this has created employment opportunities in the service sector including transport, retail, catering, and guiding jobs in the Ourika valley, but also in the oasis itself where an accommodation was built. Alternative concepts of tourism fostering sustainability and solidarity values were also created to support livelihoods in marginalized areas^55^. Such new economic opportunities did not foster agricultural production but rather resulted in the development of infrastructure and a major dependency on external resources.

Our data show a decline in labor-intensive multiple cropping patterns and a decrease in overall fertilization, in particular of manure application. Since agriculture is nowadays practiced as a secondary occupation by most households, farmers adapted their crop production to low-input farming practices. Monocropping of barley during the rainy period for feeding livestock became the main agricultural activity. This requires less labor and water compared with the multiple cropping patterns practiced before. Other studies in the region show that barley as a winter crop performs well in low input systems and does not require much protection against diseases^56^, or regular fertilizing amendment^57^. It is widely used in rainfed regimes, as it ripens faster and tolerates less water intake than other crops^58,59^. As a dual-purpose cereal it can be harvested as green fodder during years of low rainfall or as a grain in years of higher rainfall and therefore plays a major role for livestock production throughout Morocco^60,61^. Even though livestock numbers have decreased in the oasis of Tizi N’Oucheg over the past years, livestock keeping is still an important economic activity in the oasis. Its role as an instant source of cash inflow^62^ and its importance for cultural and religious events such as the *Eid Al-Adha*^63^ have been well recognized.

Decreasing precipitation attributed to climate change has been reported by many farmers as a factor determining changes in land use and agricultural activities. However, as outlined by a previous study conducted in Tizi N’Oucheg^33^, farmers’ perceptions may be better explained by the observed increase in erratic and torrential precipitation events over the past 15 years. The impact of climatic change on precipitation quantity and water flow remains somewhat uncertain, as such assessments require careful analysis of long-term meteorological data and a deeper understanding of variations in snowpacks, which serve as significant water reservoirs in the Atlas Mountains^64^. Furthermore, there is limited understanding of the total water demand for agricultural production and the relationship between precipitation levels and water storage, which replenish mountain springs. The study^65^ suggests that the decline of mountain agroecosystems may be as much a consequence of shifts in the market economy and changes in the prevailing socio-economic system as of climate change.

The production of cash crops in the oasis of Tizi N’Oucheg, such as potato and onion, did not increase over the past years despite the construction of an unpaved road which facilitates access to markets. Crop production by Moroccan smallholders faces high competition with national and international producers offering similar product quality at lower market prices. Moreover, the country’s national agricultural policies tend to favor consumers rather than supporting local smallholder production and investments in agriculture^66^.

Our study contributes to the growing body of literature on the importance of traditional agroecosystems in fostering socio-economic resilience^67,68^, conserving natural resources and biodiversity^69^, and reaching national goals of sustainability^70^. It underlines the importance of understanding the incentives of local farmers in practicing agriculture in order to find site-specific solutions. To maintain agricultural activities in Tizi N’Oucheg where agriculture is still practiced in a traditional labor-intensive way, financial incentives for farmers are needed. Certifications and labeling schemes that document and promote the quality and the origin of agricultural products from Moroccan oases such as Tizi N’Oucheg may help in accessing new niche markets. The already established tourism business can be used for marketing as part of a well-planed national strategy to avoid regional inequalities in development and overuse of natural resources.

## Conclusions

Tizi N’Oucheg is a vivid example of the widely discussed changes in mountain agroecosystems of Morocco as a consequence of disparate rural-urban development, out-migration, and the absence of effective policies supporting traditional agriculture in Morocco. Decreasing agricultural production and consequently, the demise of such agroecosystems are not only due to environmental change, as widely claimed, but more importantly to decreasing social-economic incentives for farmers to engage in traditional agriculture. Although livelihood adaptation strategies, such as the tourism sector, may be profitable in some settings, they often do not lead to the preservation of biodiversity and cultural heritage in traditional oasis systems.

## Electronic supplementary material

Below is the link to the electronic supplementary material.

Supplementary Material 1

## Data Availability

The datasets generated and/or analyzed during the current study are available from the corresponding author upon reasonable request.
