# Supplementary material for: Rural-urban transformation shapes oasis agriculture in Morocco’s High Atlas Mountains
Source: Sci Rep. 2025 Jan 27;15:3433. doi: 10.1038/s41598-024-81569-7 (PMC11772813; doi:10.1038/s41598-024-81569-7)
Supplement: Supplementary file 1 — Supplementary Material 1 [file 41598_2024_81569_MOESM1_ESM.docx]

Supplementary material 1: Headings of survey forms

**1^st^ phase of data collection – 25 farmers**

Household ID:

Name of HH head:

Number of people in the HH:

1. Socio-economic HH characteristics

Q: What is the share of crop production revenues in your total income (%)?

Q: What is the share of livestock keeping revenues in your total income (%)?

Q: What is the share of off-farm revenues in your total income (%)?

Q: What type of off-farm activities are you practicing?

1. Crop land use

Q: How much land do you own?

Q: How much land do you use for crop cultivation?

Q: Has your cropping land increased/decreased in the past 5/10/20 years?

| **Time span (years)** | **Change of crop land size (+/0/-)** | **What is the reason for change in crop land size? (Tourism/Environmental conditions/Buildings for own use/Livestock farming/ Higher sales/Other)** |
| --- | --- | --- |
| 05 |  |  |
| 10 |  |  |
| 20 |  |  |

1. Crop production
   1. *Variety*

Q: Which crops do you cultivate?

| **Species** | **Plots** | **Yield** | **Season** | **Rotation** | **% Own consumption** |
| --- | --- | --- | --- | --- | --- |
| (Annual) |  |  |  |  |  |
| (Perennial) |  |  |  |  |  |
| (Fodder) |  |  |  |  |  |

Q: Why do you cultivate these crops?

| **Crop species and importance (1-3)** | **Reason (HH consumption, Marketing, Storage ability)** |
| --- | --- |
|  |  |
|  |  |
|  |  |

Q: Have the species / varieties you cultivate changed over time? Proportions ?

| **Species/variety** | **Change** | **Since when? (mm/yyyy)** | **Reason (Labor availability/Market access/crop yield/Tourism/Other)** |
| --- | --- | --- | --- |
|  | 1 new crop |  |  |
|  | 2 increases |  |  |
|  | 3 decreases |  |  |
|  | 4 quit |  |  |

Q: Where do you get seeds from?

| **Crop** | **Origin** |
| --- | --- |
|  |  |
|  |  |
|  |  |

- 1. *Intensity/Variety*

Q: Do you use manure/compost on your fields (Y/N)?

If YES, for which crops?

| **Animal source** |  |
| --- | --- |
| **Quantity per unit area / plot** |  |
| **% Own production** |  |
| **% Bought** |  |

Q: Do you use mineral fertilizer (Y/N)?

If YES, for which crops and on which plots?

| **What kind of fertilizer?** |  |
| --- | --- |
| **Quantity? (total)** |  |
| **Price per unit** |  |
| **Time** |  |

Q: Do you irrigate your fields?

If YES:

| **What is your source of water?** |  |
| --- | --- |
| **How many days per week do you irrigate your fields?** |  |
| **Has your source of water changed within the last 20 years (Y/N)?** |  |

1. Livestock production

Q: Which animals do you rear?

| **Species** | **Total number** |
| --- | --- |
| Sheep |  |
| Goat |  |
| Cattle |  |
| Horse |  |
| Donkey |  |
| Poultry |  |
| Other (open) |  |
| Other 2 (open) |  |

Q: Which animals did you rear?

| **Species** | **20 years ago** |
| --- | --- |
| Sheep |  |
| Goat |  |
| Cattle |  |
| Horse |  |
| Donkey |  |
| Poultry |  |
| Other (open) |  |
| Other 2 (open) |  |

Q: What are the main problems in livestock husbandry?

| **Time** | **Problem 1** | **Problem 2** | **Problem 3** |
| --- | --- | --- | --- |
| **Past** |  |  |  |
| **Present** |  |  |  |
| **Future** |  |  |  |

**2^nd^ phase of data collection – 20 farmers**

Full name of spokesperson:

Full name of field owner:

Field area:

Field coordinates:

Q: Since when is the field used?

- Crop production

Crop 1:

Present status

| **Mineral fertilizers (composition)** | **Quantity (unit)** | **Organic fertilizers (animal specie)** | **Quantity (unit)** | **Frequency of irrigation** | **Yield (unit)** |
| --- | --- | --- | --- | --- | --- |
|  |  |  |  |  |  |

Past status

| **Years** | **Mineral fertilization - quantity or %** | **Organic fertilization - quantity or %** | **Irrigation - quantity or frequency** | **Yield - quantity or %** |
| --- | --- | --- | --- | --- |
| 10 years ago |  |  |  |  |
| 20 years ago |  |  |  |  |

Crop 2:

…

Crop 3:

…

Comments:
